# An unusual presentation of hypopituitarism caused by a sellar aneurysm

**DOI:** 10.20945/2359-4292-2023-0224

**Published:** 2024-04-26

**Authors:** Tijana Ičin, Kristina Stepanović, Ivana Bajkin, Nikola Boban, Dragan Anđelić, Đorđe Popović, Jovana Prodanović Simeunović, Željka Savić

**Affiliations:** 1 University of Novi Sad Faculty of Medicine Novi Sad Serbia University of Novi Sad, Faculty of Medicine, Novi Sad, Serbia; 2 Clinic for Endocrinology Diabetes and Metabolic Disorders Clinical Centre of Vojvodina Novi Sad Serbia Clinic for Endocrinology, Diabetes and Metabolic Disorders, Clinical Centre of Vojvodina, Novi Sad, Serbia; 3 Center of Radiology Clinical Centre of Vojvodina Novi Sad Serbia Center of Radiology, Clinical Centre of Vojvodina, Novi Sad, Serbia; 4 Clinic for Gastroenterology and Hepatology Clinical Centre of Vojvodina Novi Sad Serbia Clinic for Gastroenterology and Hepatology, Clinical Centre of Vojvodina, Novi Sad, Serbia

## Abstract

Hypopituitarism is a rare clinical condition that can present as a partial or complete absence of pituitary hormones. Hypopituitarism is most commonly caused by a sellar or parasellar mass, particularly a tumor, and the gold standard for its differential diagnosis is magnetic resonance imaging (MRI). Intrasellar aneurysm is an unusual cause of hypopituitarism. Indeed, about 0.17% of all cases of hypopituitarism are due to intrasellar aneurysms. We report the case of a 72-year-old man who was admitted to the hospital due to gastrointestinal symptoms and malnourishment. Due to persistent hyponatremia and spontaneous hypoglycemia in laboratory findings, the examination of the hypothalamic-pituitary-adrenal axis was eventually initiated, and the patient was later diagnosed with an unruptured aneurysm of the ophthalmic segment of the right internal carotid artery with sellar extension as a cause of panhypopituitarism. A combined endovascular treatment was performed with stent-assisted coil embolization of the aneurysm, and the patient was prescribed oral hormonal therapy. At the 1-year follow-up visit, no improvement in pituitary function was observed, and a pituitary MRI showed complete aneurysm occlusion and partial empty sella with significantly decreased pituitary volume. Aneurysms of the internal carotid artery are rare and may be associated with hypopituitarism and delayed diagnosis due to their unusual clinical presentation. Endovascular procedures, such as coil embolization of the aneurysm, could be the treatment of choice in these patients. Persistent hypopituitarism may occur even after successful treatment of the aneurysm.

## INTRODUCTION

Hypopituitarism is a rare condition caused by partial or complete inability of the pituitary gland to secrete hormones, with an estimated global incidence of 4.2 cases per 100,000 patients per year. The clinical presentation of hypopituitarism varies, as it can present as a deficiency of all pituitary hormones (*i.e.*, panhypopituitarism) or as a deficiency of individual anterior or posterior pituitary functions (1). Therefore, hypopituitarism can present with a deficiency of thyroid-stimulating hormone (TSH), growth hormone (GH), adrenocorticotropic hormone (ACTH), luteinizing hormone (LH), and follicle-stimulating hormone (FSH), in addition to central diabetes insipidus due to deficiency of antidiuretic hormone (ADH or arginine vasopressin) (2). Treatment of hypothyroidism is dependent on its etiology and is aimed at minimizing clinical signs and symptoms, usually by replacing target hormones (3). A variety of pituitary and hypothalamic disorders can result in pituitary insufficiency, but sellar and parasellar mass compression is the most common cause, with pituitary tumors estimated to cause hypopituitarism in 61% of the cases (4). Other disorders are less common and include head trauma, metastatic tumors, vascular injury, inflammation, and postpartum pituitary apoplexy (5,6). While computed tomography (CT) should be used to evaluate sellar bone anatomy, when necessary, magnetic resonance imaging (MRI) is the gold standard for the differential diagnosis of sellar and parasellar masses (7). Intrasellar aneurysm is an uncommon but well-described cause of pituitary deficiency (8,9).

Here, we report the case of a 72-year-old man who was admitted due to gastrointestinal symptoms and was later diagnosed with unruptured aneurysm of the ophthalmic segment of the right internal carotid artery (ICA) with sellar extension as a cause of panhypopituitarism.

## CASE PRESENTATION

**Figure 1:**
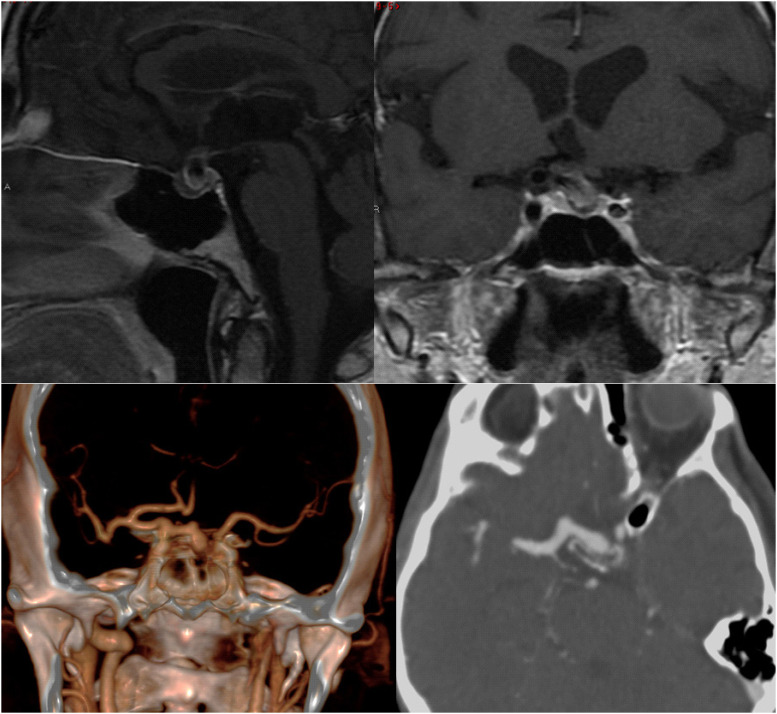
The two images on the top show a pituitary magnetic resonance image of a 10 × 11 × 8 mm sellar mass, highly suggestive of a vascular structure extending from the right internal carotid artery into the sellar region and compressing the pituitary gland. The two images on the bottom show a computed tomography angiography of the cerebral arteries revealing unruptured, partially thrombosed aneurysm of the ophthalmic segment of the right ICA, extending into the sellar region and compressing the pituitary gland.

**Figure 2:**
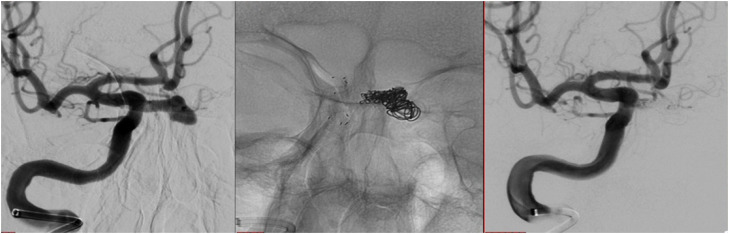
Digital subtraction angiography showing stent-assisted coil embolization of the intrasellar aneurysm.

**Figure 3:**
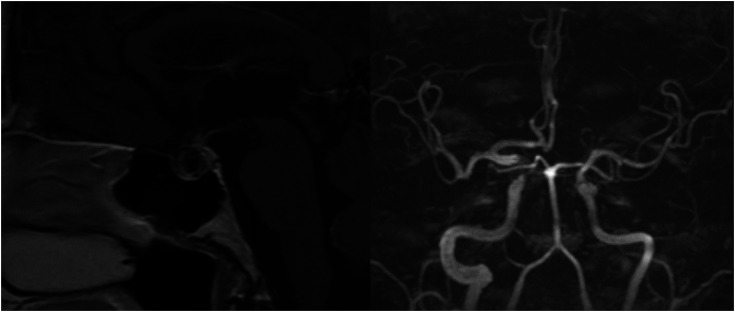
Follow-up magnetic resonance imaging (MRI) 1 year after treatment. The image on the left shows occlusion of the aneurysm and partial empty sella with substantially decreased pituitary volume. The image on the right shows a time of flight MRI with an absence of flow in most of the aneurysm sac and minor residual flow in the region of the aneurysm neck; the low signal flow in the segment of the internal carotid artery where the stent is placed is due to artifacts.

A 72-year-old man experienced nausea, abdominal pain, and weight loss of 10 kg over 4 months; thus, an outpatient colonoscopy was planned. During preparation for colonoscopy, the patient's condition worsened due to severe weakness. The colonoscopy was not performed, and the patient was admitted to the emergency department. On the day of admission, he presented with normal vital signs and a score of 15 on the Glasgow Coma Scale. The electrocardiogram was unremarkable. Physical examination revealed asthenia, malnourishment (body mass index of 18.2 kg/m^2^), epigastric pain during palpation, and traces of fresh blood on digital rectal examination. The remainder of the patient's physical examination was unremarkable. Initial laboratory results were significant for normocytic anemia, hyponatremia (122 mEq/L; reference range 135-145 mEq/L), hypochloremia (86 mEq/L; reference range 98-107 mEq/L), and increased sedimentation rate. Serum potassium levels, C-reactive protein, aminotransferases, gamma-glutamyl transferase, alkaline phosphatase, and parameters of hemostasis were within the reference range. Chest x-ray and abdominal ultrasound were unremarkable. Additional diagnostic procedures were performed. Upper endoscopy showed grade B gastroesophageal reflux disease and incompetence of gastric cardia, while abdominal CT could not rule out colon infiltration. A colonoscopy was eventually performed, revealing internal hemorrhoids, which could explain the digital rectal examination finding. Serum levels of tumor markers (serum total prostate-specific antigen, alpha-fetoprotein, carcinoembryonic antigen, carbohydrate antigen 19-9, and cancer antigen 125) were within the reference range. In subsequent laboratory tests, hyponatremia persisted despite hypertonic saline administration, and one episode of spontaneous hypoglycemia (48.6 mg/dL [2.7 mmol/L]; reference range 70.2-109.8 mg/dL [3.9-6.1 mmol/L]) was observed. These findings initiated the examination of the hypothalamic-pituitary-adrenal axis, which revealed ACTH deficiency, while additional laboratory tests showed TSH, GH, FSH, and LH deficiency with mild hyperprolactinemia (Table 1). The patient was given stress doses of hydrocortisone by intravenous infusion followed by replacement therapy with levothyroxine and progressed with significant clinical improvement. A visual field test did not reveal the typical visual field defects of chiasmal compression by a pituitary tumor. Pituitary MRI revealed a vascular structure originating from the right ICA measuring 16 × 8 × 12 mm, with an intrasellar component measuring 10 × 11 × 8 mm (anteroposterior x laterolateral x craniocaudal), highly suggestive of intrasellar aneurysm (Figure 1). Thus, CT angiography (CTA) of the cerebral arteries was performed and showed an unruptured, partially thrombosed aneurysm of the ophthalmic segment of the right ICA with sellar extension, compressing the pituitary gland and the pituitary stalk (Figure 1). Combined endovascular treatment with stent-assisted coil embolization of the aneurysm was successfully performed (Figure 2). After complete clinical recovery, the patient was discharged with maintenance doses of hydrocortisone and levothyroxine. A marginal increase in serum prolactin level indicated secondary hyperprolactinemia due to the mass effect of the aneurysm on the pituitary stalk. At the 1-year follow-up visit, no improvement in pituitary function was observed and hormonal replacement therapy was continued (Table 1). A follow-up pituitary MRI showed a near-complete aneurysm occlusion with minor residual filling in the neck region of the aneurysm and partial empty sella with significantly decreased pituitary volume (Figure 3).

**Table 1:** Endocrine panel at admission and at the 1-year follow-up visit

	Results at admission	Results at the 1-year follow-up visit*	Reference range
ACTH (pg/mL)	9	N/A	7-63
Cortisol† (nmol/L)	44.9	571.9	101.2-535.7
Cortisol‡ (nmol/L)	42.2	N/A	79.0-477.8
TSH (mIU/L)	1.73	<0.01	0.35-4.94
FT3 (pmol/L)	2.5	4.8	2.6-5.7
FT4 (pmol/L)	5.9	13.3	9.0-19.0
GH (ng/mL)	0.18	<0.05	0.110-5.500
IGF-1 (ng/mL)	12.27	30.90	184-208
FSH (IU/L)	1.03	1.45	N/A
LH (IU/L)	0.43	0.36	N/A
Testosterone (nmol/L)	0.10	<0.45	8.33-30.19
Prolactin† (ng/mL)	55.7	<0.5	5.2-26.5

* Results of adrenocortical axis and thyroid axis hormones at the 1-year follow-up were obtained during hormonal replacement therapy.

† Collected at 8 a.m.

‡ Collected at 4 p.m. Abbreviations: ACTH, adrenocorticotropic hormone; FSH, follicle-stimulating hormone; FT3, free triiodothyronine; FT4, free thyroxine; GH, growth hormone; IGF-1; insulin-like growth factor 1; LH, luteinizing hormone; N/A, not available; TSH, thyroid-stimulating hormone.

## DISCUSSION

According to the literature, intrasellar aneurysms account for 1%-2% of all intracranial aneurysms and 0.17% of all cases of hypopituitarism (10).

In a literature review conducted in 2012, Hanak and cols. identified 31 studies reporting 40 cases of intrasellar aneurysms. The authors analyzed the types of extension into the sella and divided the aneurysms into three groups: infradiaphragmatic (59% of all reported aneurysms), supradiaphragmatic (26%), and wide-neck ICA siphon aneurysms (15%) with no clear pattern of sellar invasion. Supradiaphragmatic aneurysms can originate from the ophthalmic segment of the ICA or the anterior communicating artery and have an inferomedial extension into the sella (11). Our patient presented with an unruptured, partially thrombosed, ophthalmic ICA aneurysm.

In the same study by Hanak and cols. (11), the authors concluded that the most common clinical presentation includes visual field defects/decreased visual acuity (61%), headache (61%), endocrinopathies (57%), symptomatic hyponatremia (21%), and cranial nerve (other than optic nerve) paresis (18%). The authors also noted that infradiaphragmatic aneurysms usually present with endocrinopathies, of which the most common are hyperprolactinemia and deficiency of gonadotropins, while supradiaphragmatic aneurysms are more likely to cause visual field defects. However, the available literature suggests that the clinical presentation of both supradiaphragmatic and infradiaphragmatic aneurysms may not follow the expected pattern. Qi and cols. (12) also concluded that hyperprolactinemia and hypogonadism are the most common endocrinopathies, but the patient in their report had a supraclinoid segment ICA aneurysm without the symptoms expected for a supradiaphragmatic aneurysm. Conversely, Torres and cols. (13) reported a case of a bilateral, infradiaphragmatic ICA aneurysm involving the ICA intracavernous component, extending into the sella and presenting with headache and visual field defect. In our case, the patient sought medical help due to gastrointestinal symptoms, but resolution of the symptoms after hormone treatment was initiated confirmed hypopituitarism to be the underlying cause. Despite the clinical presentation expected for supradiaphragmatic aneurysms, our patient presented predominantly with endocrinopathies and did not have visual field defects or headache. Apart from mild hyperprolactinemia and hypogonadism, our patient also had ACTH, TSH, and GH deficiency. These laboratory findings are in accordance with the ones shown by Gondim and cols., who also reported a case of an ICA aneurysm with sellar extension and panhypopituitarism with mild hyperprolactinemia (14); however, the patient in their case also presented with decreased visual acuity on the left eye, headache, and amenorrhea-galactorrhea syndrome, and the origin of the aneurysm was unclear. Similar hormonal findings have also been reported by Seok and cols. (15) in a patient who developed panhypopituitarism with mild hyperprolactinemia due to a giant ICA aneurysm with intrasellar extension; also, in this case, the origin of the aneurysm was unclear. This patient presented with hyponatremia and general weakness, as our patient did, but also had headache, as in some of the cases previously reported by other authors. Our patient did not experience headache or visual field defects.

Kageyama and cols. reported two cases of giant aneurysms with different origins; one from the cavernous sinus, in which the patient had a similar clinical presentation as our patient, with abdominal pain, weight loss, and panhypopituitarism with hyponatremia accompanied by distorted conciseness, and the other case with panhypopituitarism and hyponatremia with low-grade fever, nausea, and appetite loss as a result of an ICA aneurysm (16). The exact origin of the ICA aneurysm also was not specified (16). More recently, another case of ICA aneurysm (clinoid segment) was reported in a patient presenting with severe headache and left third nerve palsy; panhypopituitarism was diagnosed with decreased TSH, FSH, LH, testosterone, and insulin-like growth factor 1, as well as suboptimal ACTH stimulation test; no deteriorated biochemical profile was mentioned (17).

Apart from the literature data being inconsistent, the lack of a specified origin of the aneurysm in some cases further complicates the interpretation of intrasellar aneurysm presentation.

In some cases, aneurysms of the ICA, especially supraclinoid parts, may rupture and cause subarachnoid hemorrhage. Moreover, ICA aneurysms could coexist in direct contact with a pituitary adenoma. These pathologies could also lead to a diverse clinical presentation (18). Such heterogeneity in clinical presentation, the origin of the intrasellar aneurysm, and potential coexistent pathologies make each case a piece of a larger puzzle that remains to be solved.

Currently, there are several options for treating unruptured intracranial aneurysms, including surgical clipping and different endovascular procedures. Still, minimally invasive endovascular coiling is frequently used and is considered the treatment of choice (19,20). Combined endovascular treatment with stent-assisted coil embolization of the aneurysm was performed successfully in our patient.

According to a retrospective review of medical data from 1950 to 1995 that evaluated postoperative outcomes, the pituitary function remains unchanged after surgical treatment (21). Other literature data (22,23) also indicate that hypopituitarism is permanent after surgical treatment, requiring continuous hormonal replacement therapy.

Regarding the rate of pituitary function recovery after various endovascular treatments of intrasellar aneurysms, available data are inconsistent. Partial recovery of pituitary function has been observed in some cases of intrasellar aneurysms treated with an endovascular coil at 1-year (16) and 2-year follow-up visits (12). Several cases of cerebral aneurysms with hypopituitarism treated with flow-diverting stent have been reported. One of these cases showed that, at 6 months, complete functional recovery of the pituitary gland postoperatively was not achieved, though functional improvement via hormone loading test was confirmed (24). Other cases reported complete functional recovery at 3-month (18) and 10-month (23) follow-up visits, while one group of authors showed no improvement at 1 month after treatment (25); no further pituitary function assessments were reported for either of the cases. Another group of authors reported a case of a giant ICA aneurysm with pituitary dysfunction successfully treated with high-flow bypass using radial artery graft followed by ligation of the ICA at the cervical portion; at the 1-year follow-up, the patient presented with normal pituitary hormone levels, and at the 8-year follow-up visit, remained without hormonal replacement therapy, with good patency of the graft confirmed by MRI (26). According to the latest literature review, either complete or partial recovery of the pituitary function is possible (23% and 18%, respectively); unfortunately, in 59% of the cases, hypopituitarism persists (17). At the 1-year follow-up visit, no improvement in pituitary function was observed in our patient. Factors including the mass effect of the aneurysm, the extent of pituitary compression, the duration of pituitary dysfunction, and aneurysm thrombosis with subsequent inflammation are believed to influence recovery (23,24). The time to functional improvement reported ranged from 2 months to 2 years (24). The effect of various treatment options on pituitary function recovery remains unclear (12).

In conclusion, aneurysms of the ICA are rare and can potentially cause hypopituitarism. Due to unusual clinical presentation in some cases, a delay in diagnosis is possible. Endovascular procedures such as flow-diverter stent implantation and coil embolization of the aneurysm could be the treatment of choice in these patients. Persistent hypopituitarism could be expected even after successful treatment of the aneurysm, requiring continuous hormonal replacement therapy.
